# Profiles of Volatile Compounds from Seven New Hybrid Families Obtained by Crossings on Noir de Bourgogne Cultivar and Other Blackcurrant Varieties

**DOI:** 10.3390/molecules28041916

**Published:** 2023-02-17

**Authors:** Sandy Pagès-Hélary, Marine Nars-Chasseray, Laurence Dujourdy, Nathalie Cayot

**Affiliations:** 1 Institut Agro Dijon, University Bourgogne Franche-Comté, PAM UMR A 02.102, 1 Esplanade Erasme, F-21000 Dijon, France; 2 Institut Agro Dijon, Sayens Agro-Environnement, 3 Rue des Coulots, F-21110 Bretenière, France; 3 Institut Agro Dijon, Service d’Appui à la Recherche en Science des Données, F-21000 Dijon, France

**Keywords:** blackcurrant berries, cultivars, hybrids, volatile compounds, HS-SPME-GC-MS, chemo metrics, chemical profiling

## Abstract

Berries of blackcurrant (*Ribes nigrum* L.) are popular for their strong and complex aroma and their benefits for health. In Burgundy (France), the most famous blackcurrant cultivar is the “Noir de Bourgogne”. A blackcurrant breeding program was conducted to obtain new varieties, more resistant to infections and climate changes. The cultivar “Noir de Bourgogne” was crossed with seven other varieties in order to create a hybrid with good agronomic properties and organoleptic properties close to the ones of “Noir de Bourgogne”. Several hybrids were created, and their aromatic profiles studied. Berries of eight cultivars, among which Noir de Bourgogne and hybrids resulting from crossings, were harvested during the summer of 2020. Volatile compounds of berries were analyzed by HS-SPME-GC-MS, and principal component analysis (PCA) was used as the most useful chemometric technique. The profiles in volatile compounds of hybrids were either different from those of the two parental varieties or close to that of varieties other than Bourgogne black. In all cases, the overall aroma strength of the hybrid did not equal that of the Noir de Bourgogne cultivar.

## 1. Introduction

The biggest producer of blackcurrant berries is Poland [1]. In this country, berries are essentially used for juices and jams. Nevertheless, blackcurrant buds are also used in alcoholic beverages and perfumes in smaller volumes of production but are important economically [2]. Blackcurrant berries are even used as a valuable ingredient in a healthy diet [3].

Blackcurrant is a berry shrub species that has a high cold tolerance and is natively distributed in the forests of the Northern Hemisphere [4]. Quality characteristics of berries, such as berry size, soluble solids, acid, sugar and ascorbic acid content, and sugar-to-acid ratio, can be highly correlated to weather conditions and microclimate [5]. Weather conditions may also affect the organoleptic qualities of berries.

Climate change is harmful to many plants, especially berries. In fact, because blackcurrant is an early spring blooming shrub, the risk of spring frost is a matter of serious concern due to floral damage leading to poor yield. In addition, mild winters and insufficient winter chilling have profound effects on the time and evenness of bud break, time, and duration of flowering, thereby leading to considerable yield losses in susceptible cultivars [6,7]. Increasing temperatures during summer may also lead to sunburn. Processes leading to sunburn are highly complex and not fully understood, but sunburn was observed on different crops, including berries, and may be dependent on a cultivar specificity [7].

Moreover, the rise of the temperature, together with humidity, which generate the appearance of many viruses, mushrooms, and parasites as scales, leads to very poor harvests of blackcurrant, especially for the cultivar “Noir de Bourgogne” which is grown mainly in Burgundy (France) to produce “Crème de cassis de Dijon” and “Crème de cassis de Bourgogne”. This cultivar produces fruits with intense flavor and allows a liquor with the designation of origin. Unfortunately, the White Peach Scale (*Pseudaulacaspis pentagona*) is one of the most damaging armored scale insects as it is a pest of various crops in France and has become one of the main threats to blackcurrants [8].

A breeding program was then conducted in Burgundy (France) in order to generate blackcurrant plants more resistant to pests and with high heritability of the quality attributes of Noir de Bourgogne fruits. Since 2018, this breeding program has generated more than 316 hybrids from 10 parental combinations using controlled pollination methods.

Therefore, the flavor of blackcurrant berries was the main criterion, but the agronomical parameters of cultivars and hybrids were also major criteria to consider. Used agricultural criteria were primarily:

− Tolerance to diseases such as powdery mildew
− Tolerance to White Peach Scale (WPS)
− Satisfying strength and growth
− Allowed auto-pollination with flowers with stamens above the pistil
− Satisfying yield with many berries, proving the satisfying fertility
− Late blossoming to avoid frost risk.

Many desirable agronomic characteristics have been inherited by the hybrids from the tested varieties. Finally, seven varieties were kept as good candidates for crossing with the cultivar Noir de Bourgogne and gave 221 hybrid plants. The three parental combinations that were discarded from the breeding program showed poor yields and sensitivity to frost and were auto sterile.

About 204 volatile odorant compounds (VOCs) were identified in berries of selected blackcurrant cultivars [9]. Over these past three years, statistical analyses of the chromatographic data of these selected varieties of blackcurrant were made and confirmed a great demarcation of the variety “Noir de Bourgogne” for several VOCs.

Among these VOC, 20 compounds were particularly followed as previously reported as major trends for the fingerprint of Noir de Bourgogne [9]. The volatile profile of berries from the cultivar “Noir de Bourgogne” was reported to be particularly rich in 3-carene, terpinolene, and trans-beta-ocimene. Other molecules were present in small quantities but not found in the other varieties, which constitutes a unique chemical signature.

The present study is focused on blackcurrant berries used to produce French alcoholic beverages with the designation “Crème de cassis de Dijon” and “Crème de cassis de Bourgogne” [10]. The aim of the present investigation was to study the inheritance of the characteristic odorant volatile compounds of Noir de Bourgogne and other varieties by their hybrids. The first step of this study was to characterize the chemical profiles of volatile compounds for the berries of the cultivar “Noir de Bourgogne”. The second step was then to also characterize the volatile profiles for the seven other cultivars and for the hybrids to find a variety of blackcurrant enough close to the one of “Noir de Bourgogne” to replace it in the two famous Burgundy liquors. The 20 volatile compounds involved in the fingerprint of Noir de Bourgogne were semi-quantified for the berries of the other parental varieties and the hybrids resulting from the seven crossings with Noir de Bourgogne.

## 2. Results and Discussion

### 2.1. Profiles in Volatile Compounds for the Selected Cultivars

**Figure 1:**
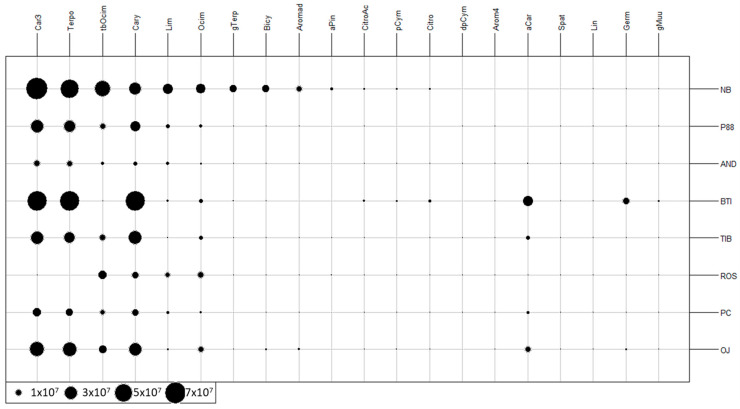
Two-dimensional table plot with proportional circles for peak areas of each volatile compound for the height varieties harvested in 2020 used in the seven crossings (NB: Noir de Bourgogne, P88: 88-04-181, AND: Andega, BTI: Ben Tiran, TIB: Tiben, ROS: Rosenthal, PC: PC110, OJ: OJ-5-3).(Car3: 3-Carene, Terpo: Terpinolene, tbOcim: trans-beta-Ocimen, Cary: Caryophyllene, Lim: Limonene, Ocim: Ocimene, gTerp: gamma-Terpinene, Bicy: Bicyclogermacrene, Aromad: Aromadendrene, aPin: alpha-Pinene, CitroAc: Citronellyl Acetate, pCym: para-Cymene, Citro: Citronellol, dpCym: dehydro-para-Cymene, Arom4: 4-aromadendrene, aCar: alpha-Caryophyllene, Spat: Spatulenol, Lin: Linalol, Germ: Germacrene D, gMuu: gamma-Muurolene).

**Figure A1:**
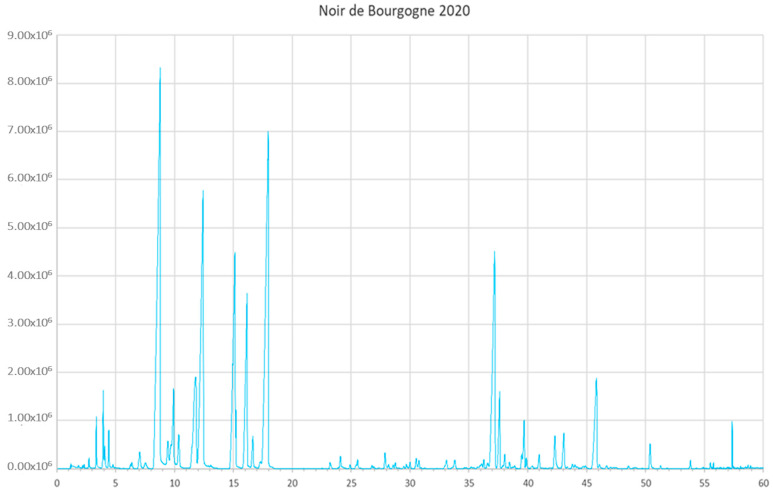
Example of chromatogram obtained for Noir de Bourgogne.

The 20 VOCs were first analyzed for parental varieties (see Appendix A, example of chromatogram in Figure A1 and mean value for concentrations in Table A1). A two-dimensional table plot with proportional circles for the peak area of each compound was shown in Figure 1 (raw data have been normalized by the weight of berries). Missing values were replaced by the detection limit value set to 50,000 (corresponding to 100,000 divided by about 2 g of sample).

The larger the circle, the higher the peak area. As expected, the cultivar Noir de Bourgogne exhibited the highest number of volatile compounds with high peak areas. Cultivar Ben Tiran also showed a higher number of compounds than other cultivars, then came the cultivar OJ-5-3 (P_OJ) and the cultivar 88-04-181 (P_88).

Caryophyllene, Limonene, and Ocimene were present in all samples but with varying amounts depending on the cultivar. Germacrene D was present only for Ben Tirran, and gamma-terpinene was present only for Noir de Bourgogne.

In order to summarize the information contained in this complex data table and to give a more accurate interpretation of the relationships between observations and variables, and among the variables a Principal Component Analysis (PCA) was undertaken.

This multivariate analysis considered the eight cultivars used for crossings and the mean values obtained for the 20 volatile compounds.

**Figure 2:**
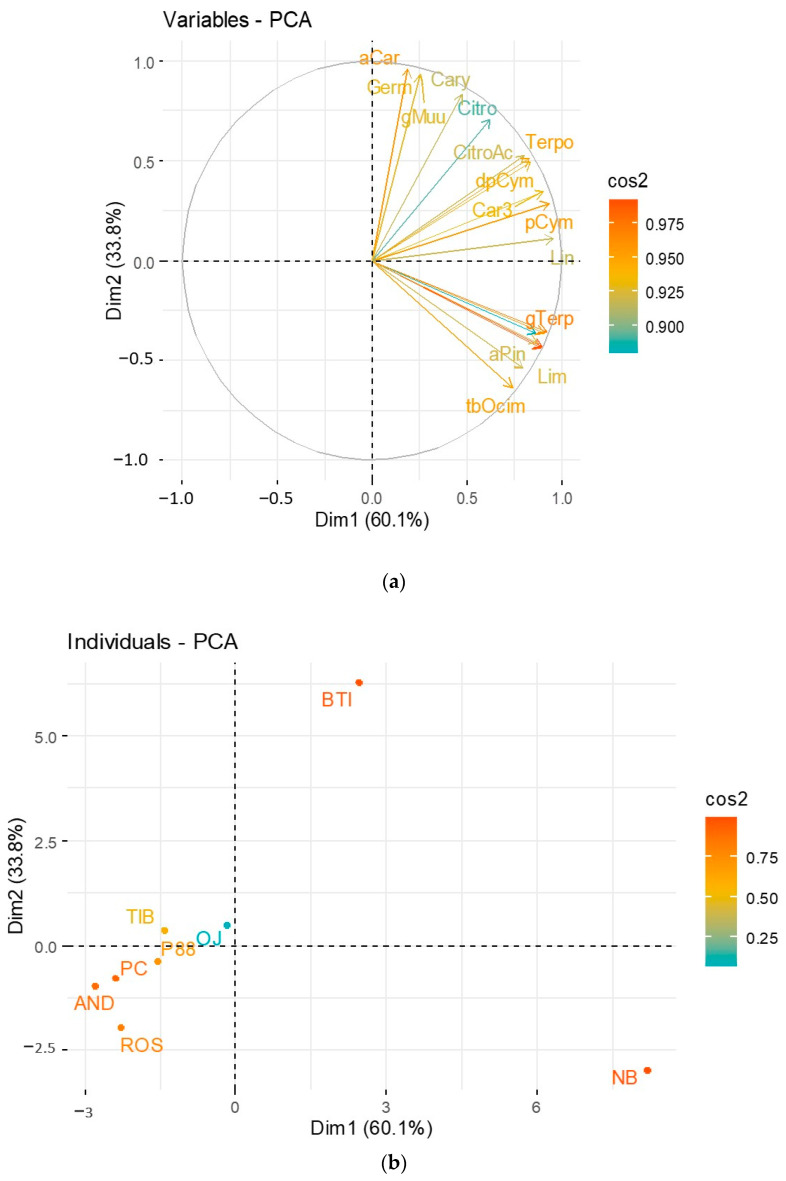
PCA plot released on the eight cultivars for the 20 molecules retained in the fingerprint of Noir de Bourgogne. Cultivars labels: Ben Tiran (BTI), Tiben (TIB), OJ-5-3 (OJ), 88-04-181 (P88), PC110 (PC), Andega (AND), Rosenthal (ROS), and Noir de Bourgogne (NB). (**a**) Representation of the variables in plan Dim1-Dim2; (**b**) Representation of the individuals (Figure 2b) on the factor map.

Figure 2a corresponded to the loadings plot in plan Dim 1-Dim 2. Figure 2b corresponded to the scores plot in plan Dim 1-Dim 2. The quality of representation of the variables (Figure 2a) and of the individuals (Figure 2b) on the factor map, called cos2 (square cosine), was given by a color gradient. A high cos2 (close to 1) indicated a good representation of the principal component.

Two dimensions accounted for 93.8% of the total variance. All variables (volatile compounds) were well represented with cos2 > 0.9.

Different groups of cultivars could be observed from the Dim1-Dim2 plan—Figure 2b). Noir de Bourgogne and Ben Tiran cultivars were far better represented than the other varieties. As seen in Figure 1, these varieties stood out from the others. As these individuals contributed the most to the PCA, the variables were represented on the right part of the plot together with Noir de Bourgogne and Ben Tiran.

Alpha-Caryophyllene, Germacrene D, and gamma-Muurolene represented the cultivar Ben Tiran. Terpinolene, dehydro-para-Cymene, 3-Carene, para-Cymene, Linalool, and gamma-Terpinene represented the cultivar Noir de Bourgogne. These specific molecules were then sought in the profiles of the hybrids obtained by crossing with the cultivar Noir de Bourgogne.

### 2.2. Dispersion of Profiles in Volatile Compounds for Different Hybrids of the Same Crossing

A given crossing led to different hybrid plants. Berries for each plant (three replicates) were collected and analyzed. It was noticeable that the profiles in volatile compounds might vary from one plant to another.

**Figure 3:**
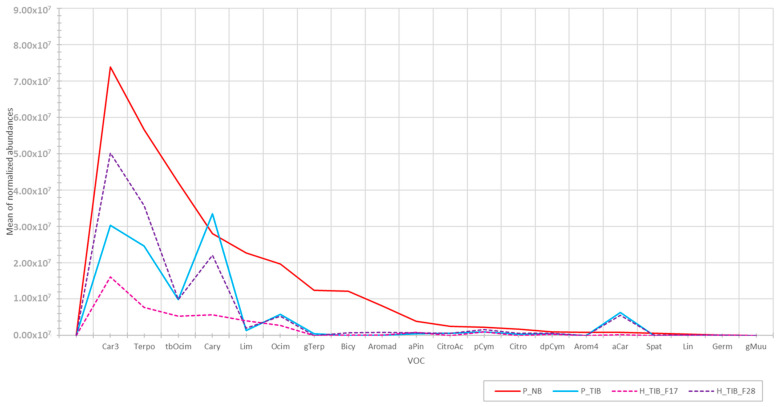
Profile in volatile compounds for Noir de Bourgogne (P_NB), Tiben (P_TIB), and two hybrid (H_TIB_F17 and H_TIB_F28) plants from their crossings (mean values of three replications).

Figure 3 shows a simplified chromatogram with only peaks of interest for two cultivars (Noir de Bourgogne and Tiben) and two hybrid plants. In this case, the profiles obtained for hybrids were quite similar.

As expected, the abundance of volatile compounds was higher for Noir de Bourgogne. The hybrid plant H_TIB_F28 exhibited a profile quite close to the one of Tiben, with similar compounds but different intensities. The hybrid plant H_TIB_F17 exhibited a profile with a shape similar to that of Noir de Bourgogne but far flatter. No hybrid plant was found to have a profile close to the one of Noir de Bourgogne.

**Figure 4:**
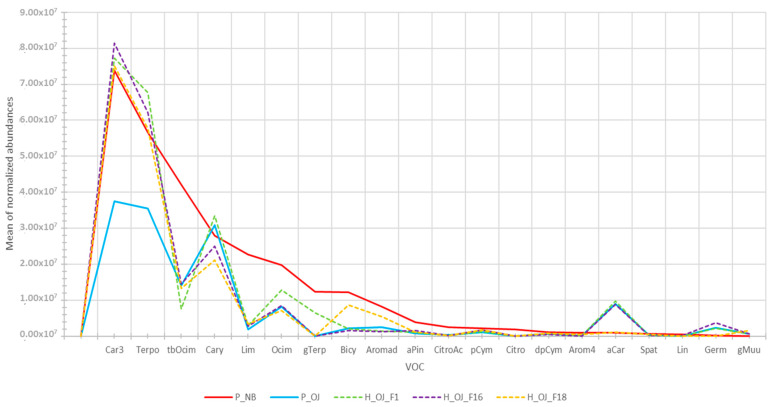
Profile in volatile compounds for Noir de Bourgogne (P_NB), OJ-5-3 (P_OJ), and three hybrid (H_OJ_F1, H_OJ_F16, and H_OJ_F18) plants from their crossings (mean values for three replications).

In the case of a crossing between Noir de Bourgogne and OJ-5-3 (Figure 4), some hybrid plants had a volatile profile with higher abundances than OJ-5-3, but still very different from the profile of Noir de Bourgogne.

### 2.3. Comparison of Volatile Compounds between Cultivars and Hybrids

The proximity with Noir de Bourgogne, on the basis of the 20 selected volatile compounds, was checked in order to study the inheritance of the characteristic aroma compounds of Noir de Bourgogne and other varieties by their hybrids.

Firstly, a PCA on cultivars and hybrids and mean values obtained for the 20 molecules was done. The hybrid OJ-5-3_F5 was removed from the PCA due to aberrant behavior.

**Figure 5:**
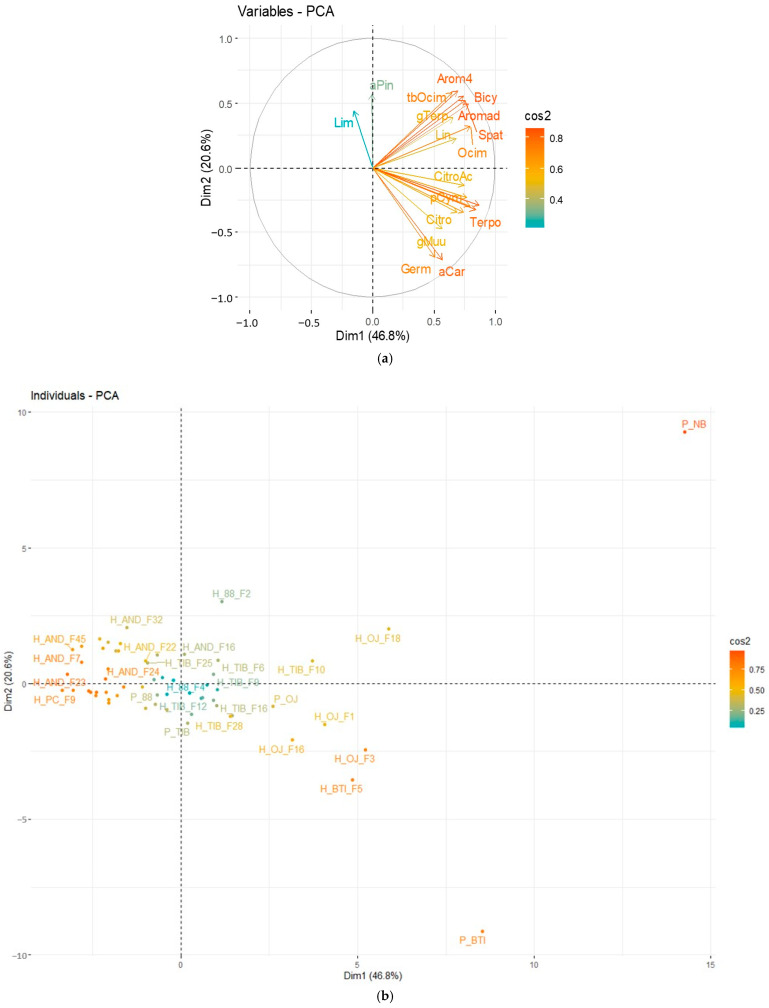
PCA plot released on the eight cultivars and hybrids for the 20 molecules retained in the fingerprint of Noir de Bourgogne (mean values). Data from berries collected in 2020; mean values, normalized using the weight of berries. Individual labels with an ‘H’ correspond to the Hybrid plants (H_Cultivar_N° of the hybrid plant), and with a ‘P’, the cultivars used in crossing to obtain these hybrid plants (P_Cultivar). (**a**) Representation of the variables in plan Dim1-Dim2 (**b**) Representation of the individuals (Figure 5b) on the factor map.

As previously explained, the color gradient represented the level of square cosines for the variables (Figure 5a) and the individuals (Figure 5b). Figure 5a corresponds to the loadings plot in plan Dim 1-Dim 2. Figure 5b corresponds to the scores plot in plan Dim 1-Dim 2.

Two dimensions accounted for 67.4% of the total variance. Some variables (Limonene and alpha-Pinene) were badly represented in this plan Dim1-Dim2 (cos2 below 0.4).

Different groups of cultivars could be observed from the Dim1-Dim2 plan—Figure 5b). Noir de Bourgogne and Ben Tiran cultivars were again far better represented than the other varieties and hybrids and very different from them.

**Figure 6:**
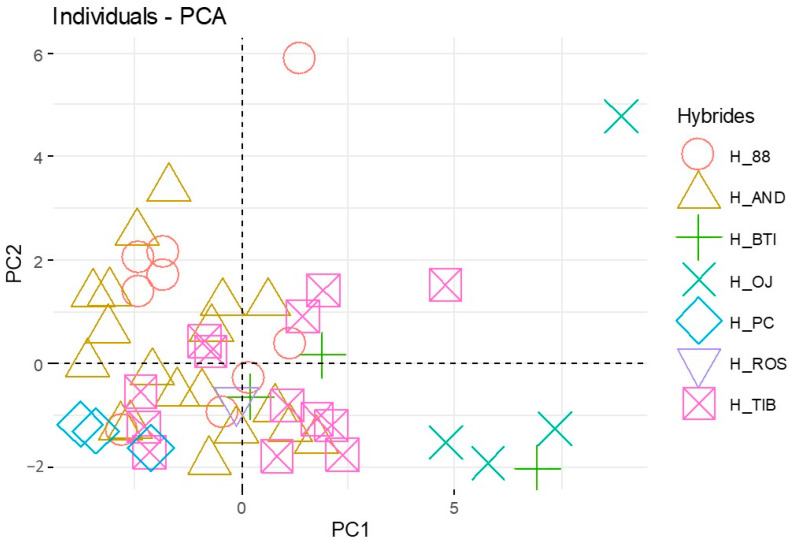
PCA plot released on the hybrids for the 20 molecules retained in the fingerprint of Noir de Bourgogne (mean values). Data from berries collected in 2020; mean values normalized using the weight of berries. Hybrid plants coming from the cross between Noir de Bourgogne and seven other varieties: 88-04-181 (H_88), Andega (H_AND), Ben Tiran (H_BTI), OJ-5-3 (H_OJ), PC110 (H_PC), Rosenthal (H_ROS), and Tiben (H_TIB).

To try to observe groups among hybrids, PCA was done only with data coming from the hybrids (Figure 6). The variables plotted in plan Dim 1-Dim 2 differed very little from Figure 5a, with no change in the interpretation of the results. Thus, we decided to show only the scores plot in plan Dim 1-Dim 2 for this PCA focused on hybrids (Figure 6). It is possible to see in Figure 6 that, even if hybrid plants are different, hybrids from the same crossing were regrouped. For instance, hybrid plants from the crossing of Andega and Noir de Bourgogne (H_AND) were all represented on the left side of the plot, with a low amount of volatile compounds.

The hybrid plants closest to Noir de Bourgogne were represented on the right side of the plot and were coming from crossing with OJ-5-3 and Ben Tiran. The hybrid plants with the lowest proximity to Noir de Bourgogne came from the crossing with PC110 and Andega. The hybrids coming from the other crossings were badly represented by the PCA, and no conclusion could be drawn.

Additionally, the dispersion of the hybrid plants was characterized using cluster analysis and a dendrogram plot (not shown). As a result, the proximity of the hybrid plants to the cultivar involved with the crossing with Noir de Bourgogne was indicated in Table 1. It was noticeable that, for all hybrid families, the profiles in volatiles were always more influenced by the parent cultivar other than Noir de Bourgogne.

### 2.4. Discussion

**Figure 7:**
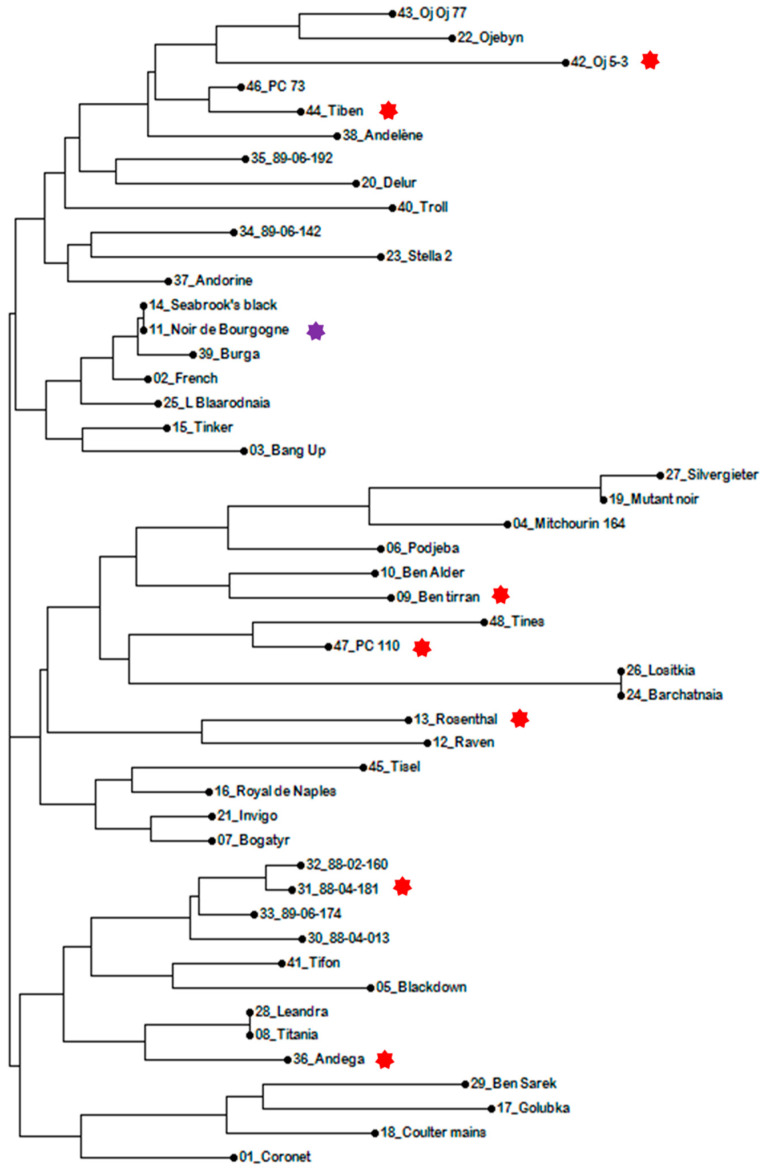
Phylogenic Tree showing similarities of some varieties used in the study. Darwin software, unweighted Neighbor joining method, using allelic data from eight SSR markers. Noir de Bourgogne cultivar was indicated with a purple star; the red stars indicated the seven other parental varieties involved in the study.

Among the different tested varieties, the cultivar Noir de Bourgogne had a profile in volatile compounds truly different from all other cultivars. The variety Ben Tiran was the closest but still very different. It is noticeable that the variety Ben Tiran originated from Scotland with a crossing with Seabrooks Black. It seems that this cultivar (Seebrooks Black) is coming from Noir de Bourgogne because it is similar to Noir de Bourgogne for all the used microsatellite markers (Figure 7).

Considering the organoleptic quality of berries and small fruits, such low degrees of inheritance were reported in other studies. For example, many experiments on small fruit breeding were conducted in Finland, among which were the breeding of strawberry and blackcurrant, and gave various results depending on the small fruit [11]. Later on, Olbricht et al. reported a combination of two cultivars of strawberry. They used data processing on 78 detectable volatile compounds and showed very high variability of the volatile patterns among hybrids. Ester methyl anthranilate was reported as a discriminative key compound, being present only in one of the mother cultivars and with a low degree of inheritance because it was found only in 25% of the hybrids [12].

## 3. Materials and Methods

### 3.1. Cultivars, Hybrids, and Cultivation

The used cultivars were mostly obtained from previous crossings with cultivars of various origins, mainly Swedish, Scottish, Polish, and French.

In the present study, cultivars were first selected on their morphologic and agronomic characteristics: the dimension of flowers, ability for self-pollination, tolerance to diseases such as powdery mildew, anthracnose, rust, and tolerance to scales.

Scores reported in Table 2 were established as follows:

− Fungal diseases: 0 = no trace to 5 = highly sensitive
− White Peach Scale: 0 = no trace to 4 = whole plant infected (on old wood, i.e., more than 2 years old)
− Flowering precocity: 1 = flowering in early April to 7 = late flowering in mid-May.
− Synchronizing of flowering: 1 = synchronous to 6 = not synchronous (meaning that different stages of flower organs were present at the same time on the plant. For the same plant, it may vary depending on the considered year. Flowering was scored weekly from April to the end of May).

A collection of 316 hybrid plants was obtained from 7 crossings between the variety of Noir de Bourgogne and other cultivars.

The selected blackcurrant cultivars (Table 2) were grown in an open field on an experimental plot located in Burgundy (France, 47°14′6.508″ N 5°6′28.096″ E). Hybrids were grown on the same plot but potted.

For cultivars, all plants were about six years old and were pruned each year in winter around January (only dead and obstructing branches were cut). Each blackcurrant plant got fertilizers (nitrogen, potassium, magnesium, and phosphorus), but neither watering nor chemical weeding. For hybrids, all plants were two years old for harvesting because they did not produce berries the first year. Only hybrids with enough berries to be analyzed were harvested and used for this study (Table 3).

### 3.2. Berry Harvesting and Storage

The eight blackcurrant cultivars were harvested at maturity. Three conditions were required to consider this fruit as ripe: berries were easy to pick, berries colored fingers when crushed between two fingers, and Brix level at harvest was over fifteen degrees. As all cultivars ripened at different times (early ripening, middle season ripening, or late ripening), the harvesting period extended for almost one month from the end of June to mid-July, as indicated in Table 2. All berries of each plant were manually picked and pooled together within the same cultivar. Cultivars gave berries of different sizes, ranging from 31 to 96 g per 100 berries, with 38 g/100 berries for the Noir de Bourgogne.

The method of harvest followed for hybrids was slightly different because there were very few berries by plant (just enough for GC-MS analyses). Therefore, it was not possible to keep berries to measure and verify the brix degree. Berries of hybrids were harvested the same day as the corresponding cultivar (other than Noir de Bourgogne). If some berries were still green, the hybrid plant was left to ripen.

Berries were stored frozen (−40 °C) until analyses for cultivars and hybrids. Storage duration ranged from one to three months before analyses.

### 3.3. Analysis of the Volatile Fraction of Berries

#### 3.3.1. HS-SPME-GC-MS

Volatiles were extracted with 1 cm SPME fiber DVB/CAR/PDMS (divi-nylbenzene/carboxen/polydimethylsiloxane; 50/30 μm). The fiber (24 Ga 50/30 µm, for the manual holder, 3 pK, 57328-U) was purchased from Sigma and used with a manual fiber holder. SPME vials (20 mL, VA201) and septum caps (18-mm caps, 8-mm PTFE/Silicon septum, SACA001) were purchased from JASCO France. About 2 g of frozen berries exactly weighed were placed in the vial. They were allowed to defrost for 30 min at ambient temperature. Preliminary experiments allowed fixing the equilibrium step at 30 min ambient temperature and the extraction step when the fiber was exposed to the headspace at 30 min ambient temperature.

An HP 6890 Series Gas Chromatograph (Hewlett-Packard, Waldbronn, Germany) equipped with an HP 5973 Mass Selective Detector (Spring, TX, USA) (Quadrupole) was used with a DB-WAX column (30 m × 0.32 mm × 0.25 µm, 123–7032, Agilent, J&W Scientific, Folsom, CA, USA) to analyze the compounds of interest. The SPME fiber was desorbed and maintained in the injection port at 250 °C for 5 min. The sample was injected with a purge flow of 20 mL/min at 2 min. Helium was used as carrier gas at 1.4 mL/min with a linear velocity of 43 cm/s. The programmed temperature was isothermal at 40 °C for 10 min, raised to 100 °C at a rate of 2 °C/min, isothermal at 100 °C for 6 min, raised to 120 °C at a rate of 4 °C/min, isothermal at 120 °C for 2 min, and then raised to 240 °C at a rate of 20 °C/min and held for 5 min. The total run time was 64 min. The ionization source and transfer line temperatures were set at 230 °C and 160 °C, respectively. The mass spectra were obtained using a mass selective detector with an electron impact voltage of 70 eV in full scan over the range *m*/*z* 29 to 400.

Analyses were done in triplicate.

#### 3.3.2. Identification of Compounds

To conduct the data analysis, compounds with an area above 100,000 (TIC intensity corresponding to peak integration threshold) and known to be odorant were kept.

Identification was based on the analysis of pure compounds: 3-Carene, γ-Terpinene, Ocimene, 1S-α-Pinene, β-Pinene, and Caryophyllene. The other volatile compounds were compared using their mass spectra in several libraries (NIST 08 (National Institute of Standards and Technology), WILEY138, and INRAMASS (personal library)). The identification of these compounds was also verified with retention index calculations.

#### 3.3.3. Selection of Representative Volatile Compounds

Compounds identified in the replicates of berries harvested in 2020 were pooled for cultivars and hybrids. For the final data analysis, the volatile compounds found at least twice on the three replicates of each sample were selected.

The compounds present in the highest amounts for cultivars and hybrids were kept. Additionally, compounds that were reported as characteristic of Noir de Bourgogne were kept.

Finally, a list of 20 molecules was traced in each sample (Table 4).

### 3.4. Chemometric Analyses

Data analysis tools can be classified as univariate, multivariate, or megavariate, depending on the number of variables considered in the analysis of a single sample at a time.

Many chemometric tools can be introduced as data projection linear methods [13], which compress raw data, uncover hidden correlations, and separate the useful information from noise. Projection methods provide a very intuitive and visual approach to data analysis, and Principal Component Analysis (PCA) is the main tool for this purpose. The subspace identified by PCA constitutes the most accurate dimensional approximation of the original data. This allows compression of the data dimensionality and, at the same time, a minimal loss of information [14].

Data analyses were carried out using the RStudio 2022.07.1 and R-4.0.5 [15,16], and specific packages: FactoMineR [17], Factoextra [18], corrplot [19], ade4 [20]. FactoMineR, factoextra, and corrplot were used for PCA, and ade4 for creating the Two-dimensional table plot.

### 3.5. Genotyping

Genotyping was done on 48 cultivars of the collection used for the breeding program. The method was adapted from Brennan et al. [21].

#### 3.5.1. DNA Extraction

DNA extraction was done using the Dneasy kit (Miniplant kit, Qiagen, Venlo, The Netherlands). The young leaves present inside the leaf buds were removed and crushed, using a pestle and Fontainebleau sand, in the kit extraction buffer. Then the extraction was carried out according to the protocol recommended by the manufacturer. The DNA solution was transferred in a fresh tube and diluted in UHQ water to get a final concentration of 5 ng/μL. Total genomic DNA concentration and nucleic acids/proteins ratio were estimated by using an Eppendorf 6131 biophotometer.

#### 3.5.2. PCR Assay and Capillary Electrophoresis

Eight microsatellites markers (SSR) were chosen: g1-K04, e1-020, e4-D03, e1-021, g2-J08, e3-B02, g2-G12, g2-L17 [21]. DNA extract (5 μL of 10 ng/μL) from each plant was added to 20 μL of a master mix [2.5 μL 10× reaction buffer (NEB), 0.5 μL 25 mM dNTPs (NEB), 0.5 μL 10 mM Primer 1, 0.5 μL 10 mM Primer 2, 0.15 μL 5 U/μL Taq DNA polymerase (NEB), and 15.85 μL UHQ water]. Forward primers are 6-FAM-labeled at the 5 end PCRs were conducted with a 2720 Thermal Cycler (Applied Biosystems, Waltham, MA, USA), with the following program: one cycle of 5 min at 95 °C, 35 cycles of 30 s at 95 °C, 30 s at 52 °C, 1 min at 68 °C, and a final extension step of 10 min at 68 °C. PCR products were screened by capillary electrophoresis using the ABI 3730 XL (Applied Biosystems) of the GENTYANE Genotyping and Sequencing platform (Clermond-Ferrand, France). Electrophoregrams were analyzed with the Gene-Mapper software (Applied Biosytems). A phylogenic Tree was drawn using the DARwin software, with the Unweighted Neighbor joining method.

## 4. Conclusions

The qualitative and quantitative differences in volatile composition were observed in a population of blackcurrant cultivars and hybrids. About 200 volatile compounds were identified and semi-quantified identified using mass spectra. Twenty of them were considered as characteristics for the odorant profile of blackcurrant berries obtained with the cultivar Noir de Bourgogne and were traced for other varieties.

In the present study on blackcurrant, by comparing the organoleptic profile of hybrids and of both parental cultivars, it seems that most of the time, the organoleptic profiles of hybrids are closer to the parental cultivar other than the Noir de Bourgogne or different from both parental cultivars.

Hybrids coming from the same crossing had organoleptic profiles different from the Noir de Bourgogne, sometimes close to one of the other cultivars used for the crossing of Noir de Bourgogne.

The hybrids with an organoleptic profile the most similar to the one of Noir de Bourgogne were the hybrids coming from the crossing with the variety of OJ-5-3.

Different siblings of the same crossing showed different volatile compositions, implying complex genetic controls for aroma-volatile production. These volatile differences among hybrids provide fundamental information for improved genetic understanding and future improvement.

## Figures and Tables

**Table 1:** Dispersion of the profiles in volatile compounds among the different hybrids.

Crossing	Hybrid Plants Close to the Cultivar Crossed with Noir de Bourgogne	Hybrid Plants Different from the Two Cultivars Used for the Crossing
Noir de Bourgogne × OJ-5-3	H_OJ_F1, H_OJ_F3, H_OJ_F16	H_OJ_F18
Noir de Bourgogne × Tiben	H_TIB_F12, H_TIB_F16, H_TIB_F22, H_TIB_F27, H_TIB_F28	H_TIB_F2, H_TIB_F17, H_TIB_F24, H_TIB_F25, H_TIB_F29
Noir de Bourgogne × Andega	H_AND_F38, H_AND_F48	H_AND_F7, H_AND_F10, H_AND_F16, H_AND_F18, H_AND_F20, H_AND_F22, H_AND_F23, H_AND_F24, H_AND_F29, H_AND_F30, H_AND_F31, H_AND_F32, H_AND_F34, H_AND_F35, H_AND_F39, H_AND_F45, H_AND_F46
Noir de Bourgogne × PC110	H_PC_F1, H_PC_F6, H_PC_F9	
Noir de Bourgogne × Ben Tiran		H_BTI_F5, H_BTI_F6, H_BTI_F11
Noir de Bourgogne × 88-04-181	H_88_F16, H_88_F18	H_88_F2, H_88_F4, H_88_F6, H_88_F9 H_88_F12, H_88_F22, H_88_F27
Noir de Bourgogne × Rosenthal		H_ROS_F4

**Table 2:** Description of cultivars used for crossings.

Cultivar	Country of Origin	Presence of Rust	Presence of Powdery Mildew	Presence Antracnose	Presence of White Peach Scales (WPS) Pseudaulacaspis Pentagona	Flowering Precocity	Synchronizing of Flowering	Weight of Berries for 3 Plants (Grams)	Date of Harvest	Weight of 100 Berries (Grams)
Noir de Bourgogne	France	1	1.5	3	2.5	5	6	848	24 June	38
Andega	France	1	0	1.5	0.5	4	3	1956	23 June	70
Tiben	Poland	0	0	2	0.5	4	5	5144	29 June	78
Oj 5-3	France	0	0	1.5	0	5	6	2353	30 June	70
Ben Tiran	Scotland	2	0	0	0	7	5	1888	16 July	76
Rosenthal	Germany	0	0	0	1	5	4	488	25 June	31
PC 110	Poland	0	0.5	1	0.5	4	3	3188	22 June	96
88-04-181	France	2.5	0	2	0	3	3	3113	25 June	58

**Table 3:** Hybrids obtained in 2018, harvested in 2020, and involved in the present study.

Crossings	Number of Harvested Hybrid Plants * among the Total Number of Grown Plants Resulting from Crossing	Labels of Harvested Hybrids
Noir de Bourgogne × OJ-5-3	4 among 32	H_OJ_F1, H_OJ_F3, H_OJ _F16, H_OJ_F18
Noir de Bourgogne × Tiben	13 among 37	H_TIB_F2, H_TIB_F6, H_TIB_F9, H_TIB_F10, H_TIB_F12, H_TIB_F16, H_TIB_F17, H_TIB_F22, H_TIB_F24, H_TIB_F25, H_TIB_F27, H_TIB_F28, H_TIB_F29
Noir de Bourgogne × Andega	19 among 37	H_AND_F7, H_AND_F10, H_AND_F16, H_AND_F18, H_AND_F20, H_AND_F22, H_AND_F23, H_AND_F24, H_AND_F29, H_AND_F30, H_AND_F31, H_AND_F32, H_AND_F34, H_AND_F35, H_AND_F38, H_AND_F39, H_AND_F45, H_AND_F46, H_AND_F48
Noir de Bourgogne × PC110	3 among 34	H_PC_F1, H_PC_F6, H_PC_F9
Noir de Bourgogne × Ben Tirran	3 among 26	H_BTI_F5, H_BTI_F6, H_BTI_F11
Noir de Bourgogne × 88-04-181	9 among 35	H_88_F2, H_88_F4, H_88_F6, H_88_F9, H_88_F12, H_88_F16, H_88_F18, H_88_F22, H_88_F27
Noir de Bourgogne × Rosenthal	1 among 20	H_ROS_F4

* Hybrids which had enough berries to be harvested and analyzed in HS-SPME-GC-MS.

**Table 4:** List of the 20 molecules involved in the fingerprint of Noir de Bourgogne and measured in the study.

CAS Number	Molecules	Codes	Odor Descriptor
80-56-8	α-Pinene	aPin	WOODY, PINE, CAMPHOREOUS, HERBAL, TERPENIC, earthy, tropical
3779-61-1	Trans-β-Ocimen	tbOcim	Sweet, herbal
99-85-4	γ-Terpinene	gTerp	TERPENIC, TROPICAL, CITRUS, LIME, *OILY*, *green*, *fruity*, woody, sweet
150-84-5	Citronellyl acetate	CitroAc	Citrus, lime, dirty, aldehydic
13466-78-9	3-Carene	3Car	CITRUS, TERPENIC, PINE, HERBAL, RESINOUS, phenolic, medicinal, spicy
99-87-6	P-Cymene	pCym	TERPENIC, WOODY, CITRUS, SPICY, ORIGANUM, *rancid*, *pepper bell*, *pepper*, fresh, cumin, oregano, cilantro
1195-32-0	Dehydro-p-Cymene	dpCym	SPICY, MUSTY, CLOVE, GUAIACOL, NUTTY, *balsamic*, phenolic, styrene, coffee
138-86-3	Limonene	Lim	CITRUS, HERBAL, TERPENIC, *pine*, *minty*, *woody*, camphorated
6753-98-6	α-Caryophyllene	aCar	woody
502-99-8	Ocimene	Ocim	GREEN, WOODY, TROPICAL, terpenic, vegetable
586-62-9	Terpinolene	Terpo	Fresh, *terpenic*, *herbal*, *floral*, WOODY, sweet, pine, CITRUS
112421-19-9	4-Aromadendrene	Arom4	not determined
78-70-6	Linalool	Lin	FLORAL, CITRUS, WOODY, WAXY, blueberry, terpenic, aldehydic
87-44-5	Caryophyllene	Cary	SPICY, WOODY, CLOVE, dry, nutty, powdery, peppery, skin
489-39-4	Aromadendrene	Aromad	Woody
30021-74-0	g-Muurolene	gMuu	Herbal, woody, spice
37839-63-7	Germacrene D	Germ	Woody, spicy
24703-35-3	Bicyclogermacrene	Bicy	Green, woody, weedy
106-22-9	Citronellol	Citro	FLORAL, citrus, green, waxy, terpenic
6750-60-3	Spatulenol	Spat	Earthy herbal fruity

Descriptors were taken from www.thegoodscentscompany.com (accessed on 21 October 2022). Descriptors in normal letters are for odor, in italics for flavor, and in capital letters for both odor and flavor.

## Data Availability

Data is contained within the article.

## Sample Availability

Samples of the compounds are not available from the authors.

## Appendix A

**Table A1:** Retention time, retention index, and mean values of the relative concentration of three replications for the 20 molecules followed in all parental cultivars.

CAS	Molecules	RT	RI	MA	SD
80-56-8	α-Pinene	3.9	991	1.29 × 10^6^	1.10 × 10^6^
3779-61-1	Trans-β-Ocimen	14.9	1219	1.28 × 10^7^	1.30 × 10^7^
99-85-4	γ-Terpinene	15.0	1206	1.94 × 10^6^	4.23 × 10^6^
150-84-5	Citronellyl acetate	42.3	1655 *	8.84 × 10^5^	1.23 × 10^6^
13466-78-9	3-Carene	8.4	1135 *	3.26 × 10^7^	2.54 × 10^7^
99-87-6	P-Cymene	16.5	1228	1.08 × 10^6^	6.59 × 10^5^
1195-32-0	Dehydro-p-Cymene	27.8	1461 **	5.14 × 10^5^	3.89 × 10^5^
138-86-3	Limonene	11.6	1157	6.19 × 10^6^	6.91 × 10^6^
6753-98-6	α-Caryophyllene	41.3	1687 **	5.57 × 10^6^	7.44 × 10^6^
502-99-8	Ocimene	15.9	1240 **	7.23 × 10^6^	5.60 × 10^6^
586-62-9	Terpinolene	17.1	1242	2.84 × 10^7^	2.22 × 10^7^
112421-19-9	4-Aromadendrene	33.0	1513 ***	1.65 × 10^5^	3.02 × 10^5^
78-70-6	Linalool	36.3	1514	1.37 × 10^5^	1.36 × 10^5^
87-44-5	Caryophyllene	37.0	1543	2.59 × 10^7^	1.82 × 10^7^
489-39-4	Aromadendrene	37.4	1556	1.52 × 10^6^	2.80 × 10^6^
30021-74-0	g-Muurolene	42.7	1638	3.49 × 10^5^	6.47 × 10^5^
37839-63-7	Germacrene D	43.7	1652	1.74 × 10^6^	3.71 × 10^6^
24703-35-3	Bicyclogermacrene	45.7	1675	1.91 × 10^6^	4.20 × 10^6^
106-22-9	Citronellol	50.4	1728	9.05 × 10^5^	1.34 × 10^6^
6750-60-3	Spatulenol	57.4	2057	1.58 × 10^5^	2.18 × 10^5^

Legend: RT (retention time in min); RI (DB-Wax retention index in min); MA (standardized mean area for the eight parent cultivars); SD (standard deviation of the mean). * [22] ** [23] *** [24].
